# Current challenges in different approaches to control COVID-19: a comprehensive review

**DOI:** 10.1186/s42269-022-00730-2

**Published:** 2022-03-03

**Authors:** Simran Giri, Sanjukta Sen, Rohan Singh, Paramita Paul, Ranabir Sahu, Gouranga Nandi, Tarun Kumar Dua

**Affiliations:** Department of Pharmaceutical Technology, University of North Bengal, Raja Rammohunpur, P.O.- NBU, District- Darjeeling, West Bengal 734013 India

**Keywords:** COVID-19, SARS-CoV-2, Coronavirus, Pandemic, Respiratory, Airborne, Treatment of COVID-19

## Abstract

**Background:**

The World Health Organization declared the outbreak of the novel coronavirus (COVID-19) as a global health emergency on January 30, 2020, and as a pandemic disease on March 11, 2020. This review highlights the international situation, risk factors, and related protections to be taken as prerequisite measures and probable treatment options for the COVID-19-infected population in the current scenario.

**Main text:**

The SARS-CoV-2 viruses and their variants caused mild-to-severe respiratory tract infection and used airborne pathways as a way of contagion. Human-to-human transmission led to an exponential growth in the rise in the number of cases making it a real burden to immobilize the rapid spread of the virus while asymptomatic patients created ambiguity for confirmation in the community. It was clear from the case studies of patients that most of them were asymptomatic but still vulnerable to the people around, and hence, in a flash, many countries around the globe went into a complete lockdown, influencing the economy and thrashing industrial outputs. On the other hand, numerous researches were made to counteract the spread through studies in antiviral therapy, immune-based therapy, vaccination development, and natural remedies.

**Conclusion:**

Although exploration for a specific drug required for the COVID-19 treatment is under extensive research worldwide and some of them are in clinical trial now. Virtual drug library screening is one of the current techniques for repurposing accessible compounds. This review could provide beneficial information about the potential current and future treatment strategies to treat the pandemic COVID-19 infection.

## Background

Severe acute respiratory syndrome coronavirus 2 (SARS-CoV-2) is the coronavirus strain responsible for the novel coronavirus 2019 (COVID-19) infection. COVID-19 emerged in the Wuhan city of China in late 2019 and swiftly occupied many of the individuals across the city with symptoms of atypical pneumonia, resulting in an outbreak of epidemic phase (Zhu et al. 2020a, b). Later, World Health Organization (WHO) declared the outbreak as a pandemic after assessing the situation around the world on March 11, 2020 (Cucinotta and Vanelli 2020). By the end of July 2021, more than 192 million cases had been recorded worldwide, resulting in the deaths of more than 4.1 million individuals worldwide from the beginning of the pandemic (WHO 2022a). Evidence till now supports the statement that COVID-19 has a zoonotic source as a constructed virus would have shown genomic sequences of known elements. The other forms of coronavirus include severe acute respiratory syndrome coronavirus (SARS-CoV) and middle East respiratory syndrome coronavirus (MERS-CoV), which has severe effects on the respiratory health of humans, while human coronavirus stains such as (HCoV) HKU1, NL63, OC43, and 229E show mild symptoms (Chilamakuri and Agarwal 2021; Corman et al. 2018). Throughout the COVID-19 pandemic, SARS-CoV-2 genetic variants have emerged and spread across the world. Airborne transmission is a major concern of SARS-CoV-2 as the expiratory activities (i.e., coughing, sneezing) of an infected person can generate respiratory droplets and infect individuals within a radius of 6ft (Ghinai et al. 2020). The front portion of the mouth is where atomization of droplets occurs; thus, covering the mouth by the use of a surgical facemask is essential (Johnson et al. 2011).

The incubation period of a virus is the period between the exposure and the potential earliest date of symptoms, and current research showed that the incubation period for COVID-19 ranges from 2 to 7 days while the median estimate is 4 days (Guan et al. 2020). Fever, dry cough, dyspnea, myalgia, tiredness, regular or reduced leukocyte counts, and radiographic indications of pneumonia are all common signs of COVID-19 infection. Symptoms of lung abnormalities, lymphopenia, and thrombocytopenia have also been observed in certain COVID-19 individuals. The pathophysiological feature of COVID-19 is governed by proliferative and exudative stages of alveolar damage, necrosis of pneumocytes, inflammatory infiltrates, and microvascular damage (Carsana et al. 2020). To successfully combat current and possible future pandemics, detailed investigations of this new coronavirus, its mode of infection, and replication are required. This review focuses on the global situation, clinical manifestation, precautions to be taken as a precautionary measure, and various treatment approaches for COVID-19.

## Main text

### History and classification

Coronaviruses are a family of hundreds of viruses, and it was seen that the majority of these viruses showed their harmful effect on different animals like bats, chickens, camels, and cats. In the 1960s, human coronaviruses 229E and OC43 were first discovered that were able to infect humans (Andersen et al. 2020). Among human coronaviruses, four are endemic (229E, OC43, NL63, and HKU1) and are well known for causing mild diseases (Kahn and McIntosh 2005; Saxena et al. 2020).

In November 2002, the first SARS-CoV virus was identified, resulting in severe acute respiratory syndrome (SARS) (Lau et al. 2020). In 2003, the members of Canada's National Microbiology Laboratory identified this virus's genome and confirmed the reason for this outbreak (Pal et al. 2020). Since 2005, several novel coronaviruses have been recognized from bats, and the evidence showed that human respiratory coronaviruses, SARS coronavirus, and MERS coronavirus were initially derived from bat viruses ancestral (Paden et al. 2018; Burrell et al. 2017).

Another deadlier coronavirus MERS-CoV (Middle East respiratory syndrome) was discovered in 2012. In MERS, the first case was from Saudi Arabia. Later another two MERS outbreak was identified in 2015 and 2018 in South Korea and Saudi Arabia, respectively. Then the first SARS-CoV-2 or COVID-19 infection was reported in December 2019 in Wuhan city of China. The virus was first discovered in bats and then pangolins (Panyod et al. 2020; Zhang et al. 2020). The genomic structure of virus SARS-CoV and SARS-CoV-2 bears many common characteristics and shows almost similar symptoms. This disease turns life-threatening if people are suffering from SARS. A total of 774 people was died from 2002 to 2014, according to the last reported case (Abdul-Fattah et al. 2021).

On account of their genus, there are four main subgroups of coronaviruses, known as alpha (*α*), beta (*β*), gamma (*γ*), and delta (*δ*) coronaviruses. Among them *α* and *β* coronaviruses are known to infect mammals, and other two *γ* and *δ* coronaviruses are known to create infection on birds (Wertheim et al. 2013; Guo et al. 2020).

### Structure

Coronaviruses are large, roughly spherical, and consisting of particles with bulbous surface projections (Goldsmith et al. 2004). It is a single-stranded RNA-enveloped virus with the largest genomes (26.4–31.7 kb) among all known RNA viruses belonging to the *Coronaviridae* family. The virus envelope appears to be two separate electron-dense shells in electron micrographs (Huang et al. 2020; Mousavizadeh and Ghasemi 2020; Fehr and Perlman 2015).

**Fig. 1:**
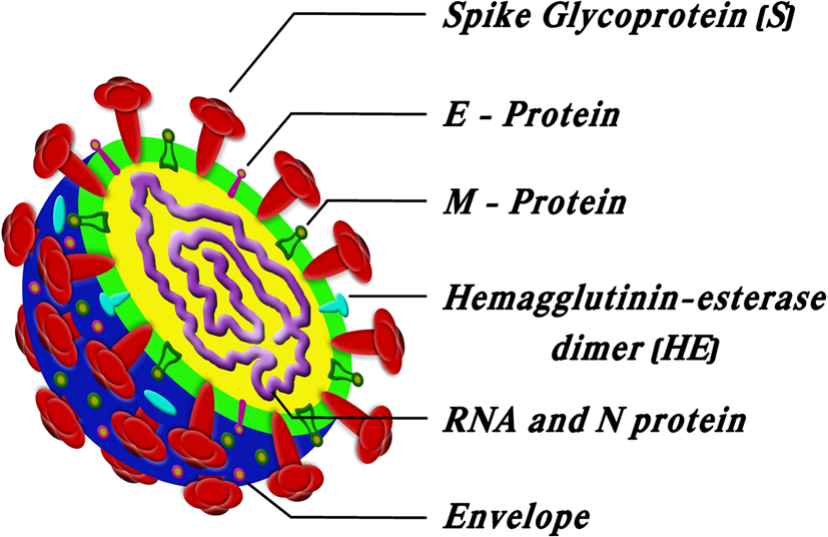
Structure of coronavirus

The viral envelope of coronaviruses consists of three main structural proteins. These are the very large glycoprotein S (for spike), glycoprotein M (membrane protein), and the internal phosphorylated nucleocapsid protein (N), and there is another minor transmembrane protein E also present (Fig. 1) (Burrell et al. 2017; Huang et al. 2020). Each type of protein in the virions of coronaviruses shows different characteristics (Table 1). Moreover, some coronaviruses further contain a shorter spikelike surface protein termed hemagglutinin esterase (HE) (Haque et al. 2020). The surface spikes of coronavirus are homotrimers of the protein S. It comprises two subunits S1 and S2, which are associated with cell recognition and the combination of viral and cellular membranes, respectively (Coutard et al. 2020). The S1 subunit takes place at the head of the spike, and there is a receptor-binding domain (RBD). The S2 subunit creates a stem that attaches to the viral envelope's spike (Huang et al. 2020). The nucleocapsid (N) protein is linked to positive-sense single-stranded RNA in a continuous beads-on-a-string type fabric. The M proteins and nucleocapsid protect the virus skeleton when placed outside the host cell, which is responsible for the shape of the virion (Mousavizadeh and Ghasemi 2020; Agrahari et al. 2021).

**Table 1:** Characteristics of different types of structural protein (Haque et al. 2020; Huang et al. 2020; Boopathi et al. 2021; Mousavizadeh and Ghasemi 2020)

Type of structural protein	Characteristics
Nucleocapsid protein (N)	N-protein coats the viral RNA genome which plays a vital role in its replication and transcription. It is responsible for encapsulating and protecting (+)-RNA, which contains the virus genome
Spike protein (S)	Type I membrane glycoprotein is made of 1160–1400 amino acids S-protein facilitates viral entrance into the host cell by mediating receptor-binding and membrane fusion between the virus and the host cell
Envelope protein (E)	E-protein is a tiny membrane protein with 76–109 amino acids that is a minor component of the viral particle. It is involved in virus assembly, host cell membrane permeability, and virus–host cell contact
Membrane protein (M)	The M-protein is most prevalent on the viral surface and is thought to be the coronavirus's major organizer. It neutralizes the virus-specific antibodies that have formed inside the host cell Determines the shape of the viral envelope
HE protein	The HE protein may have a role in viral entrance; it is not necessary for virus replication, but it appears to be important for natural host cell infection

### SARS-CoV-2 variants

SARS-CoV-2 is susceptible to genetic evolution, causing several variants with different features than their ancestral strains. Several variations of SARS-CoV-2 have been reported throughout this pandemic, of which only a handful are considered variants of concern (VOCs) by the WHO, considering their propensity to produce greater transmissibility or virulence, reduction in neutralization by antibodies obtained via natural infection or vaccination, the capacity to avoid detection, or a decline in treatments or vaccine efficacy (Cascella et al. 2022; WHO 2022b). Variants having particular genetic markers that have been linked to alterations are known as variants of interest (VOIs). The Centers for Disease Control and Prevention (CDC) and WHO have developed a categorization method for separating SARS-CoV-2 variations into VOCs and VOIs. An updated list (Table 2) of VOCs and VOIs has been prepared by WHO to assist in establishing monitoring and research priorities (WHO 2022b).

**Table 2:** List of SARS-CoV-2 variants of concern (VOCs) and variants of interest (VOIs) (Cascella et al. 2022; WHO 2022b)

Name of the variant	Lineage	First reported	Date of the first report
*Variants of concern (VOCs)*			
Alpha	B.1.1.7	UK, September 2020	December 18, 2020
Beta	B.1.351	South Africa, May 2020	December 18, 2020
Gamma	P.1	Brazil, November 2020	January 11, 2021
Delta	B.1.617.2	India, October 2020	VOI: April 4, 2021 VOC: May 11, 2021
Omicron	B.1.1.529	South Africa, November 2021	November 26, 2021
*Variants of interest (VOIs)*			
Epsilon	B.1.427/B.1.429	USA, March 2020	March 5, 2021
Zeta	P.2	Brazil, April 2020	March 17, 2021
Eta	B.1.525	Multiple countries, December 2020	March 17, 2021
Theta	P.3	Philippines, January 2021	March 24, 2021
Iota	B.1.526	USA, November 2020	March 24, 2021
Kappa	B.1.617.1	India, October 2020	April 4, 2021
Lambda	C.37	Peru, August 2020	June 14, 2021
Mu	B.1.621	Colombia, January 2021	August 30, 2021

### Global situation

**Fig. 2:**
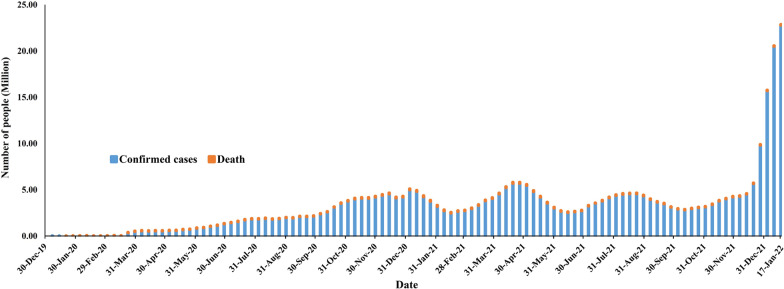
Total number of confirmed cases and deaths globally at the end of every week from its emergence

COVID-19 confirmed cases were increased within a few months from its emergence, then by the mid of February 2021, confirmed cases were reduced to some extent, and in April and August 2021, confirmed cases have increased. In January 2022, confirmed cases increased enormously. As per the WHO report, by January 25, 2022, there have been 364,191,494 confirmed cases of COVID-19, including 5,631,457 deaths. According to the WHO report, the world's confirmed cases and mortality with time during this pandemic has been explained with a graphical presentation in Fig. 2 (WHO 2022a).

### Clinical manifestations

**Fig. 3:**
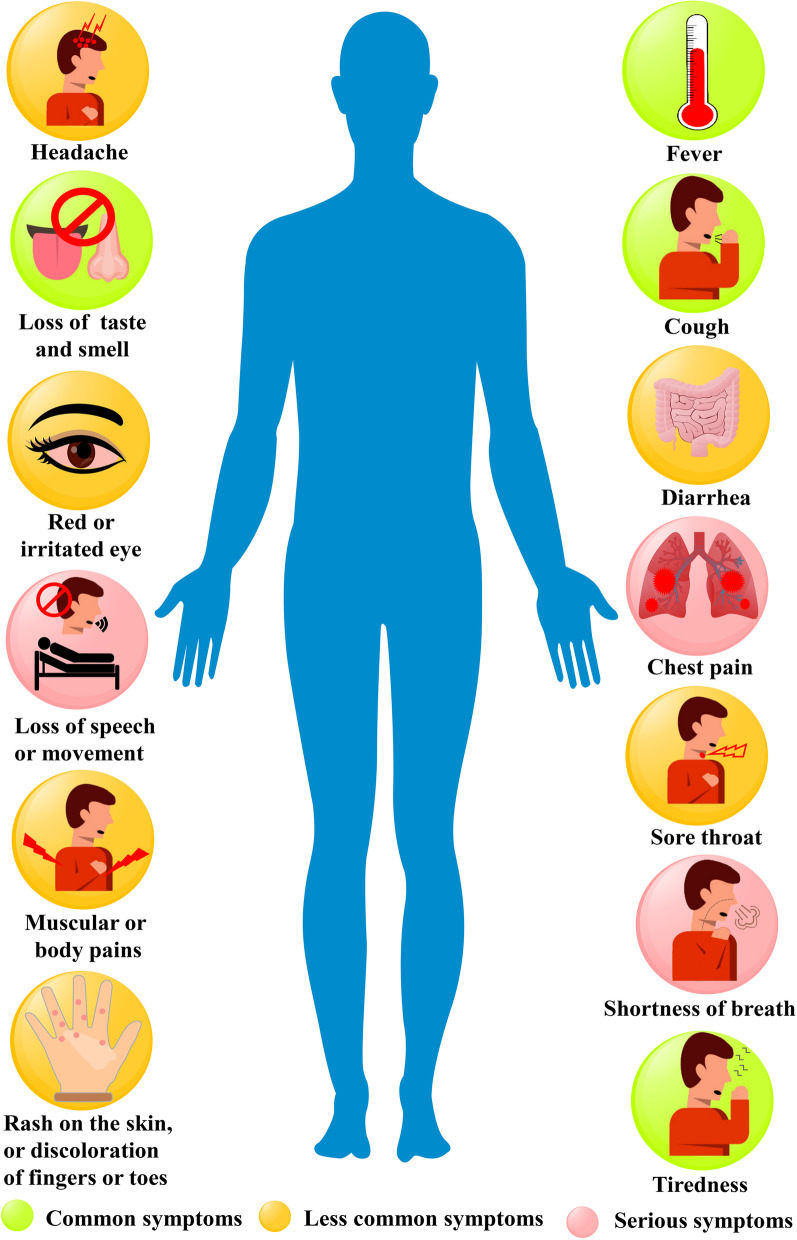
Different symptoms of COVID-19 patients

COVID-19 is a contiguous disease and is transmittable from an infected person to healthy individuals via air droplets, and the virus can affect different people in peculiar ways. Mild-to-moderate illness occurs in the infected people, while some recover without prior hospitalization. The most prevalent symptoms of COVID-19 patients are fever, muscular or body pains, dry cough, loss of taste or smell, myalgia or tiredness, pneumonia, and complex dyspnea, and less common symptoms are designated by aches and pains, sore throat, diarrhea, conjunctivitis, headache, skin rash, discolouration of fingers or toes, nausea, and vomiting. Breathing difficulties, shortness of breath, chest discomfort or pressure, and loss of speech or movement are all severe symptoms. Different symptoms of COVID-19 patients have been graphically represented in Fig. 3. Most symptoms appear 2–14 days after virus exposure in most symptomatic patients (Huang et al. 2020). For patients with health comorbidities, such as cardiovascular illnesses, renal impairment, liver dysfunction, diabetes, Parkinson's disease, and cancer, both young and old individuals' health might get complicated. Most people with mild-to-moderate illness may recover without requiring special treatment. Within 2–4 weeks of therapy, healthy people may recover from the viral illness (Harcourt et al. 2020).

### Preventions

**Fig.4:**
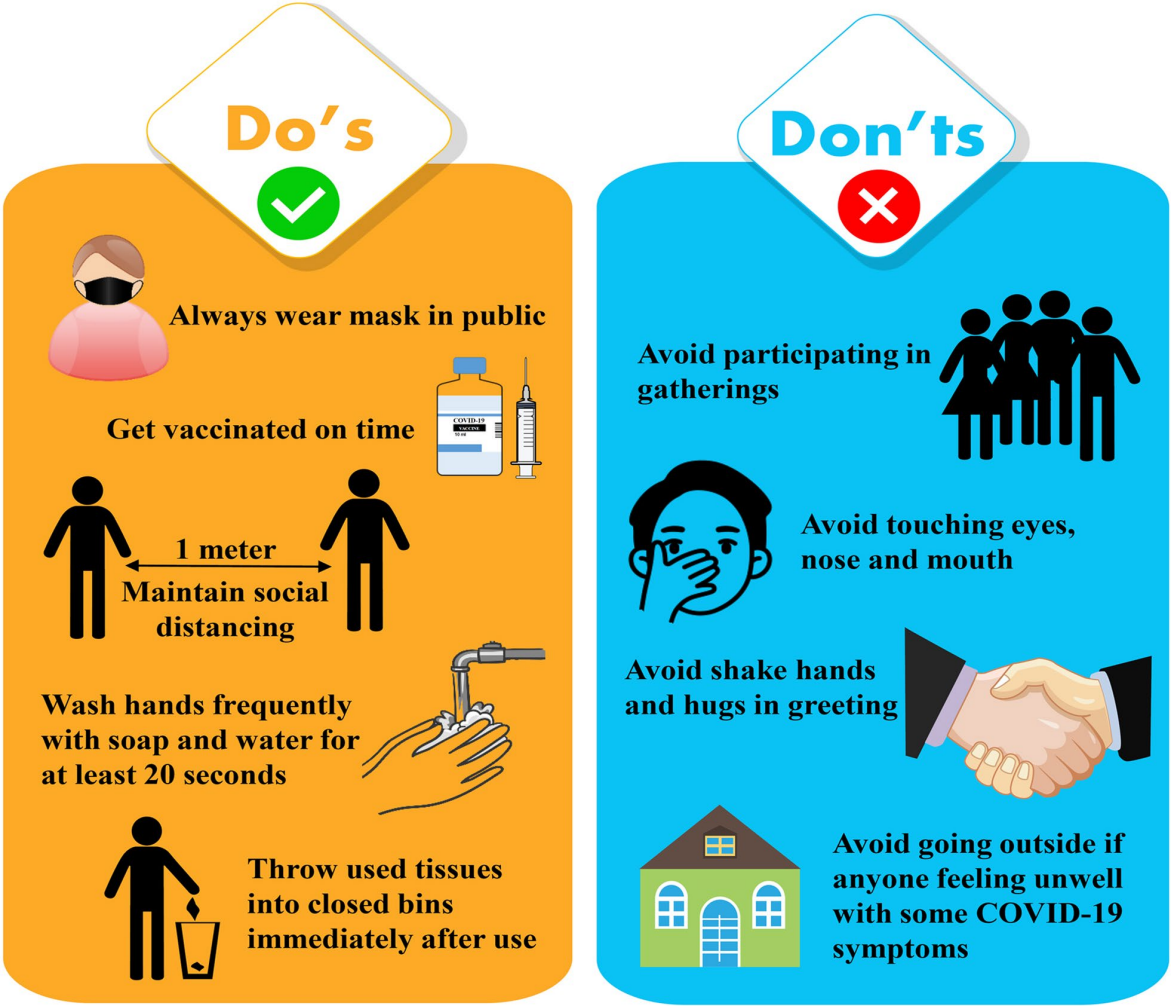
Preventive measures to control COVID-19 infection

Prevention is better than cure; hence, the spread of this disease can be controlled by paying constant attention to some basic preventative measures (Fig. 4) given below.

- Get vaccine on time, and follow the local vaccination guideline.
- Maintain social distancing of at least 1-m space between yourself and others to reduce your threat of infection when others cough, sneeze, or talk (Huang et al. 2021).
- Use a face mask when being around other people (Clapham and Cook 2021).
- Frequently, in a proper manner, clean and rub your hands (at least 20 s.) with an alcohol-based hand wash or usage soap, followed by rinsing with water. If hand wash or soap is not available, use alcohol-based hand sanitizer (minimum 60% alcohol).
- Avoid going to crowded, congested, and/or involving close touch areas.
- Surfaces that are often handled, such as doorknobs, faucets, and phone displays, should be cleaned and disinfected regularly.
- Cover the nose and the mouth with the bent elbow or tissue during coughing or sneezing. After that, throw away the used tissue in a closed container and wash hands to maintain good respiratory hygiene.
- If anyone is feeling unwell with some COVID-19 mild symptoms, they should stay home and self-isolate until they recover. If the person develops fever, cough, or difficulty breathing, get medical treatment, call the doctor ahead of time if possible, and follow your local health authority's instructions (WHO 2022c).

### Present treatments of COVID-19

Since the beginning of the COVID-19 pandemic, different measures have been taken to treat COVID-19 infection. Still, there is no clinically validated and specific antiviral medication available to treat COVID-19 infection at this time. Patients are usually provided medical treatment or supportive therapy, such as oxygen supplementation and mechanical ventilation, to alleviate symptoms (Cucinotta and Vanelli 2020). For the treatment of COVID-19 infection, many strategies have been explored, and repurposing drugs is one of them. Some of the antivirals that have been repurposed include remdisivir, lopivir, lopinavir–ritonavir, ribavirin, baloxavirmarboxil, favipiravir, and arbidol/umifenovir. Other drugs that show potential action against COVID-19 but are not antivirals include chloroquine, hydroxychloroquine, corticosteroids, losartan, statins, interferons, nitric oxide, and epoprostenol, which are instances for the repurposing strategy. Some medications have been suggested for treatment in critically sick patients. Tocilizumab, siltuximab, sarilumab, anakinra, and ruxolitinib were used to treat COVID-19 individuals who developed cytokine release syndrome (CRS). Antibiotics like azithromycin are frequently used to treat secondary infections (Ginsburg and Klugman 2020). The vaccine is another better approach for the prevention of COVID-19 infection. Though various vaccines against the SARS-CoV-2 virus have recently been approved, availability remains a major barrier, and public acceptance has become a contentious issue. But day by day, SARS-CoV-2 virus becomes more contagious and harmful due to their new mutants called variants. Recently the omicron variant's capacity to evade vaccine-elicited immunity is a major concern. So, there is a requirement for potential therapeutic molecules to treat the infection. Several antiviral drugs might be potentially repurposed or developed into viable treatments for this novel coronavirus. However, several clinical trials exploring possible therapies are now underway.

#### Remdesivir

Remdesivir is a broad-spectrum antiviral drug with antiviral efficacy in vitro against SARS-CoV-2 by inhibiting viral replication (Wang et al. 2020). According to Holshue et al. (2020), the first instance in the USA (reported in Washington State) was treated with an intravenous injection of remdesivir, which alleviated symptoms and had no adverse effects. In randomized controlled clinical studies, remdesivir was shown to be superior to placebo in decreasing the time to recovery in patients hospitalized with mild-to-severe COVID-19 infection (Beigel et al. 2020; Goldman et al. 2020). Remdesivir was approved by US Food and Drug Administration on October 22, 2020, for the treatment of COVID-19 necessitating hospitalization in adult and children patients 12 years old and weighing a minimum of 40 kg (US FDA 2021).

#### Favipiravir

Favipiravir is a broad-spectrum antiviral and anti-influenza drug that restricts viral RNA replication by inhibition of RNA polymerase (Fang et al. 2020). Several studies have revealed that favipiravir can effectively treat COVID-19, particularly in patients with mild-to-moderate disease. Favipiravir has been demonstrated in certain investigations to lower viral load in the upper respiratory tract and the lungs (Shirali and Daikoku 2020). Additional well-designed trials, including therapy dosage and duration investigations, are required before conclusive findings can be reached (Manabe et al. 2021).

#### Lopinavir–Ritonavir

It is an FDA-approved antiretroviral medication for HIV disease that was recommended as an antiviral treatment for COVID-19 during the early stages of the pandemic. A randomized control study in individuals hospitalized with severe COVID-19 found no benefit from lopinavir–ritonavir therapy compared to the standard of care (Cao et al. 2020).

#### Arbidol

Arbidol is also known as umifenovir, a Russian antiviral drug that seems to be effective against many viruses, including influenza, respiratory syncytial virus (RSV), poliovirus, rhinovirus, Zika virus, hepatitis and SARS-CoV, and MERS-CoV coronaviruses (Gao et al. 2020). The trimerization of SARS-CoV-2 spike glycoprotein, which is important for cell adhesion and penetration, may be effectively blocked or hampered by arbidol. When the trimerization of the SARS-CoV-2 spike glycoprotein is blocked, a bare or immature virus is formed, which is less infectious (Vankadari 2020). As a host-targeting drug, Arbidol also disrupts many steps of viral cycle replication, including entrance, attachment, internalization, and membrane fusion. Arbidol substantially improved the clinical state of hospitalized COVID-19 patients, including peripheral oxygen saturation, the need for ICU admissions, the length of hospitalization, chest computed tomography involvements, white blood cells, and erythrocyte sedimentation rate, according to a randomized controlled trial (Nojomi et al. 2020).

#### Ivermectin

Ivermectin is a broad-spectrum antiparasitic drug that the FDA has authorized. Based on an in vitro research that indicated the reduction of SARS-CoV-2 replication, it was utilized globally to treat COVID-19. Later on, a randomized control study including 476 adult patients with moderate COVID-19 disease could not produce substantial improvement or remission of symptoms after being randomized to take ivermectin for 5 days or a placebo (Caly et al. 2020).

#### Chloroquine and hydroxychloroquine

Chloroquine and hydroxychloroquine have a long history of use in the inhibition and treatment of malaria and the treatment of chronic inflammatory disorders such as rheumatoid arthritis. Hydroxychloroquine is derived from chloroquine, and initially, they have been proposed as an antiviral treatment for COVID-19 (Gasmi et al. 2021; Horby et al. 2020). Later on, a randomized controlled trial reported that hydroxychloroquine was administered as a postexposure prophylactic within 4 days of a high-risk or moderate-risk COVID-19 exposure, and hydroxychloroquine did not protect against COVID-19-related illness or infection (Boulware et al. 2020; Mitjà et al. 2021). Another open-label randomized controlled trial reported that chloroquine/hydroxychloroquine treatment in patients brought to the hospital with severe COVID-19 resulted in clinical deterioration and increased rates of invasive mechanical ventilation and renal failure (Réa-Neto et al. 2021). The Food and Drug Administration (FDA) granted emergency use authorization (EUA) to chloroquine and hydroxychloroquine in May 2020 to treat severe cases of COVID-19 in hospital settings. The FDA, however, removed the emergency use authorization for chloroquine and hydroxychloroquine on June 15, 2020, in situations where clinical studies were lacking (Omokhua-Uyi and Staden 2021).

#### Anakinra

Anakinra is a 17-kD recombinant human IL-1 receptor antagonist (blocking both IL-1α and IL-1β), with a short half-life of around 3–4 h and a favorable safety profile, authorized for the treatment of rheumatoid arthritis, gouty arthritis, and other uncommon auto-inflammatory disorders. Anakinra is a safe and effective treatment strategy for delaying mechanical ventilation, reducing the need for supplemental oxygen, and regulating SARS-CoV-2-triggered inflammation in patients with severe COVID-19 pneumonia and a high oxygen need (Balkhair et al. 2021). Later on, Tharaux et al. reported that the patients with COVID-19 and mild-to-moderate pneumonia, a randomized clinical study found that anakinra was ineffective in lowering the requirement for noninvasive or mechanical ventilation or mortality. More research is needed to determine the effectiveness of anakinra in additional categories of individuals with more severe COVID-19 and in different settings (Tharaux et al. 2021).

#### Tocilizumab

Tocilizumab, a monoclonal anti-interleukin-6 (IL-6) antibody, has been identified as a possible therapeutic option for COVID-19 patients at risk of cytokine storms. IL‐6 is an essential cytokine in inflammatory reaction and immune response and is one of the most significant cytokines involved in COVID‐19-induced cytokine storms (Luo et al. 2020). However, a different study report showed that it is an effective treatment preference for critically ill COVID-19 patients, as it substantially reduces their oxygen requirements and their ICU stay, median hospital stay, and death. COVID-19-induced cytokine storms are effectively treated with this drug by decreasing the level of IL-6 (Chachar et al. 2021; Luo et al. 2020).

#### Sotrovimab

Sotrovimab, a monoclonal antibody, has been developed to treat various types of coronaviruses, including COVID-19. It is primarily used to treat mild and moderate COVID-19 infection and prevent the progression of the disease condition from critical to severe. A retrospective study reported that the use of sotrovimab significantly improved symptom resolution, outcome, laboratory marker, and decreased hospitalization rate in individuals with mild and moderate COVID-19. This study suggests the use of sotrovimab in the early stages of COVID-19 treatment (Elesdoudy 2021). Later on, Guta and his group reported that the sotrovimab lowered the probability of disease progression in high-risk patients with mild-to-moderate COVID-19. And there were no threatening signs found during the study (Gupta et al. 2021). The use of sotrovimab for treating mild or moderate COVID-19 in patients at high risk of hospitalization has also been conditionally recommended by the WHO (Kmietowicz 2022).

#### Dexamethasone

Dexamethasone is a glucocorticoid drug used to treat COVID-19 infection. In hospitalized patients with COVID-19 and respiratory failure who needed supplementary oxygen or mechanical ventilation, a low dose of dexamethasone (6 mg daily for 10 days) reduced death. Data also suggest that mortality may be higher in hospitalized patients who do not receive oxygen (Matthay and Thompson 2020).

#### Ruxolitinib

Ruxolitinib is an inhibitor of JAK 1 and 2 used to treat myelofibrosis (Wang et al. 2020). JAK inhibitors reduced the need for invasive mechanical ventilation and improved survival in people with COVID-19, most significantly baricitinib (Chen et al. 2021). Ruxolitinib, a JAK inhibitor, is under phase III clinical trial of patients with COVID-19 associated with cytokine storm and acute respiratory disorder syndrome (Valenzuela-Almada et al. 2021).

#### Baricitinib

Baricitinib is a Janus kinase (JAK) 1 and 2 inhibitor used to treat rheumatoid arthritis (Cantini et al. 2020). One of the most usually prescribed medicines for COVID-19-related pneumonia is baricitinib. WHO also recommends it for the treatment of COVID-19 (Kmietowicz 2022). It is a well-known medication with a strong affinity for infected cells (Richardson et al. 2020). According to statistics, creatine kinase in patients who took bacitracin surpassed the allowed threshold. Alternatively, high creatine kinase levels might make it difficult to start baricitinib therapy (Praveen et al. 2020). A randomized, double-blind, placebo-controlled study found that combining the baricitinib with remdesivir to treat hospitalized patients with COVID-19 pneumonia was safe and preferable to remdesivir alone. The confluence was associated with a lower risk of severe side effects (Kalil et al. 2021).

#### Convalescent plasma therapy

Convalescent plasma treatment is a type of adoptive immunotherapy that can treat a wide range of illnesses. Passive immunity can be created by using antiviral antibodies from recovered individuals to treat additional patients with a specific infectious illness. Other respiratory viral diseases, including SARS-CoV-1, H1N1 influenza, MERS-CoV, West Nile virus, and Ebola virus, have recently been treated with this technique (Marano et al. 2016). Hyperimmune immunoglobulin showed a statistically significant reduction in the risk of death among those treated with convalescent plasma or serum in all of the investigations (Al-Tawfiq and Arabi 2020). This treatment has played an essential role in treating COVID-19 patients when no effective antiviral drugs are available. In an initial uncontrolled case series, five critically sick patients with COVID-19 and acute respiratory distress syndrome underwent convalescent plasma treatment, and the result showed improvement in their clinical status (Shen et al. 2020). Later on, Duan et al. (2020) reported that a single dosage of CP (200 mL) was well tolerated and could considerably raise or sustain neutralizing antibodies at a high level, resulting in viremia disappearing in 7 days. However, clinical symptoms improved quickly over 3 days. This suggests that CP might be a viable rescue strategy for severe COVID-19 and that a randomized study is necessary. Due to sample and experimental design constraints, a definitive conclusion on the potential efficacy of this form of treatment cannot be made, and further clinical observations will be required.

#### Azithromycin

Antibiotic azithromycin belongs to the macrolide family used to treat a wide range of Gram-positive bacterial infections. By interfering with protein synthesis and mRNA translation, it inhibits bacterial growth. This antibiotic is useful in the treatment of bacterial pneumonia. In addition to its antibacterial properties, it possesses immunomodulatory and anti-inflammatory properties, which may help to reduce the difficulties caused by respiratory viral infections such as COVID-19 (Pani et al. 2020; Echeverría-Esnal et al. 2021; Oldenburg and Doan 2020). However, according to the meta-analysis study by Mangkuliguna et al. (2021), in COVID-19 patients, azithromycin did not provide significant clinical improvement, although it was well tolerated and safe to use. Because of the absence of therapeutic advantages, azithromycin should be avoided to use routinely unless bacterial pneumonia is present. So, further clinical studies are required to elucidate the role of azithromycin in COVID-19 treatment.

#### Vaccines

The world has taken different significant actions to control the COVID-19 pandemic from its beginning. However, the disease spreads unabated, wreaking havoc on people's health, social lives, and economies. Therefore, the prevention and control of the COVID-19 pandemic were immediately needed. Several vaccines have been studied, produced, tested, and assessed at a breakneck speed, and in 2021 many vaccines have been approved. According to the WHO, more than 9.8 billion vaccination doses had been delivered as of January 27, 2022 (WHO 2022a). The immune system is triggered by vaccination, resulting in the generation of neutralizing antibodies against SARS-CoV-2. But the variants of the virus are always a major cause of concern. Vaccines have long been known to lose their potency over time. As a result, various countries have authorized the administration of an additional dose of vaccine (known as a booster) to people 3–5 months after their vaccination cycle is completed. This method appears to be efficient in preserving SARS-CoV-2 immunity. Some important vaccines of the world are listed in Table 3.

**Table 3:** Some approved COVID-19 vaccines with their developer, origin, type, dosage and storage conditions

Name of the vaccine	Developer	Developer country	Vaccine type	No. of doses	Efficacy (%)	Storage (°C)	References
BNT162b2 (Comirnaty)	Pfizer-BioNtech	USA and Germany	mRNA	IM (2)	95	− 80 to – 60	Polack et al. (2020)
mRNA-1273 (Moderna COVID-19 Vaccine)	Moderna	USA	mRNA	IM (2)	94.0	− 25 to − 15	Baden et al. (2021)
Ad26.COV2.S (Janssen COVID-19 vaccine)	Janssen Pharmaceuticals	USA and Germany	Viral vector	IM 1	81.7	2–8	Sadoff et al. (2021)
ChAdOx1 nCoV-19 (AZD-1222) (Covishield)	Oxford-AstraZeneca	UK, Sweden and India	Viral vector	IM (2)	81	2–8	Voysey et al. (2021)
Sputnik V	Gamaleya Research Institute	Russia	Viral vector	IM (2)	91.6	2–8	Logunov et al. (2021)
BBV152 (Covaxin)	Bharat Biotech	India	Inactivated	IM (2)	78	2–8	Ella et al. (2021)
CronaVac	Sinovac	China	Inactivated	IM (2)	50.4	2–8	Zhang et al. (2021)
BBIBP-CorV	Sinopharm	China	Inactivated	IM (2)	79.3	2–8	Xia et al. (2021)
NVX-CoV2373	Novavax	USA	Protein subunit	IM (2)	96	2–8	Shinde et al. (2021)
CVnCoV	CureVac	Germany	mRNA	IM (2)	48.2	2–8	Hadj Hassine (2021)
AD5-nCOV (Convidecia)	CanSino Biologics	China	Viral vector	IM, IN (1)	63	2–8	Wu et al. (2021)
SCB-2019	Clover Biopharmaceu ticals	China	Protein subunit	IM	67	2–8	Bravo et al. (2022)
ZyCoV-D	Cadila Healthcare	India	DNA plasmid	ID (3)	66.6	2–8	Chavda et al. (2021)
ZF2001 (Zifivax)	Anhui Zhifei Longcom	China	Protein subunit	IM (3)	82	2–8	Yang et al. (2021)

*IM* intramuscular, *IN* intranasal, *ID* intradermal

## Conclusions

Extensive research has been being carried out on SARS-CoV- 2 and its different variants to combat them with the new treatment strategies. With the continued enormously hard efforts to prevent the spread of SARS-CoV- 2 globally, we hope that this pandemic disease will subside in the coming couple of months like SARS and MERS. Some strategies like vaccination, social distancing, self-quarantine, stay home, stay safe, night curfew, partial or complete lockdown, maintaining hygiene, wearing masks, and using hand sanitizer frequently have been imposed to control the transmission of COVID-19. Moreover, parallel clinical trials have been conducted on the proposed therapeutic options, including vaccines and potent drugs repurposed for COVID. The global availability of COVID-19 vaccines has been compensated for and overcome by international collaborations and competitions among pharmaceutical industries and researchers in scientific institutions, starting with the historically fast clinical trials. To produce safe and effective vaccines, various technologies have been used. Presently, multiple vaccines have been approved, which are significantly efficacious toward prevention of COVID-19. Some of them are also showing promising results against newer strains. This review would help the readers to understand the current scenario of the COVID-19 pandemic.

### Future prospective

The pandemic situation by SARS-CoV-2 is still wreaking havoc all over the world due to the rapid spreading ability of the virus, the lack of risk assessment, and the inclination to precipitate severe illness in co-morbid conditions. The associated enhancement in the mortality rate is genuinely an alarming public health issue. Disease control has become more complex with the increasing number of COVID-19 cases per day, particularly because no effective drugs against COVID-19 are being identified. In the current review, we presented a brief overview of SARS-CoV-2 infection ongoing treatment options based on the significant recent findings on some therapeutic strategies that could be used for future treatment of SARS-CoV-2 infection. Nonetheless, additional advanced research in the area and clinical studies would reveal the significance of the findings of the current review.

## Data Availability

Not applicable.

## Abbreviations

WHO: World Health Organization
COVID-19: Novel coronavirus
US FDA: US Food and Drug Administration
SARS-CoV-2: Severe acute respiratory syndrome coronavirus 2
SARS-CoV: Severe acute respiratory syndrome coronavirus
SARS: Severe acute respiratory syndrome
MERS: Middle East respiratory syndrome
HE: Hemagglutinin esterase
RBD: Receptor-binding domain
VOCs: Variants of concern
VOIs: Variants of interest
CDC: Centers for Disease Control and Prevention
CRS: Cytokine release syndrome
